# Supplementary material for: Impact of lower limb osteoarthritis on health-related quality of life: A cross-sectional study to estimate the expressed loss of utility in the Spanish population
Source: PLoS One. 2020 Jan 24;15(1):e0228398. doi: 10.1371/journal.pone.0228398 (PMC6980637; doi:10.1371/journal.pone.0228398)
Supplement: Attachment — Submitted filename: Reviewers_reponses_AC.docx [file pone.0228398.s003.docx]

Reviewer Comments: **Impact of lower limb osteoarthritis on health-related quality of life: An estimate of the loss of expressed utility in the Spanish population.**

**Reviewer’s summary:** The study looks at HRQL decreases associated among OALL patients compared to the general population without OALL from the Spanish National Population Survey in 2011-2012. The main outcomes of the study are two measures of HRQL from the EQ-5D-5L; the objective index score and the subjective VAS.

Overall, the study is well conceptualized with clearly articulated aim and methods. The statistical models employed are consistent with the outcomes of interest. Using the EQ-5D provides a unique advantage that the authors have not leveraged. Of particular note, the authors should consider discussing their findings in light of the clinical relevance based on Minimally Clinically Important Differences (MICD) that have been well documented for the EQ-5D versions. This is especially important for potential interpretation and policy implications of the study findings.

Other minor, albeit important comments are listed below.

**Materials & Methods:**

1. Line 128 simply starts with the phrase: “Cross-Sectional Study”. If the intention of the authors is to indicate that the study design is a cross-sectional study, they should do so by making a complete statement. Please check the abstract for a similar
2. Line 162: The use of “Chronic diseases diagnosed” should be clarified. For instance, the authors may consider to list the number of chronic diseases included. Adjusting for concurrent chronic diseases is important in HRQL studies. However, even more important is the specific chronic conditions that are accounted for in the model.
3. Line 169 – 171, the author indicates that the WOMAC was used. Is it to be assumed that the English version was used? If a Spanish version was used, kindly provide more information about the version such as its construct validity and reliability (with references).

**Results:**

1. Table 1: The multiplicity of p values < 0.0001 is an indication of a high likelihood of having type 1 error, potentially because of the large sample size. The author should consider accounting/adjusting for type 1 error in the study.
2. The author has not defined Social Groups I to VI that are used in all tables. It is hard to tell which a higher social group is and which is lowest.
3. Line 213: The use of older people (except for extreme ages) is not immediately obvious. The author should kindly indicate what age group(s) or ranges you are referring to.
4. Table 4:

- In the BMI category, the author should consider changing “normal” to “Non-obese”, given that underweight/overweight/obese people are also “normal”.
- The author included BMI and number of chronic diseases in their final model. Research indicates that multiple chronic diseases do cluster in obese populations. Was there a correlation between BMI and number of chronic diseases? If so, could there be an issue of collinearity?

5. Line 251: The authors indicate that being treated in a “hospital environment is associated with an average decrease in utility..” What may be the reason for this findings? Is it possible that the reduced HRQL associated with hospitalization is because of less than quality care in the hospital environment? Or is it as a result of the fact that more severe cases of OALL are likely to be hospitalized? Kindly provide more details.

**Discussion:**

1. Line 254: The statement “*This is the first study in Spain to quantify the utility loss attributed to OALL*….” is not consistent with the study aim to evaluate the loss due to OALL (line 122). The authors should consider rephrasing the statement.
2. The statement “*OALL is associated with a substantial decrease in the utility attributed to the state of health when this association is adjusted for other potentially confounding variables”* is ambiguous at best. HRQL research using the EQ-5D usually qualifies quantitative decreases in the utility index as statistical or clinical differences based on a defined minimally clinical important difference (MCID). The authors should consider discussing their findings within the parameters of MCID. This has the potential for interpretation especially for clinical application.
3. The authors discussed “utility” as their outcome, contrary to the fact the HRQL was the outcome in the stated aim. Please consider re-phrasing related statements for clarity and consistency.
4. Given the plethora of tests across each sub-group of the patient population and outcomes, the authors should consider adjusting for multiple testing or at least provide a rationale if they choose not to.
5. The authors should discuss limitations of using the EQ-5D-5L and its potential implications on interpretation of the outcome (for instance a ceiling effect due to the upper limit of the score).

**Implications:**

Line 337: “These results provide quantification of the prevalence of the disease and the burden..” It is not clear what the authors refer to as the “prevalence of the disease”. The aim of the study was not to present the “prevalence” of the disease.
